# Epidermolysis bullosa acquisita improved with use of dupilumab

**DOI:** 10.1016/j.jdcr.2026.05.011

**Published:** 2026-05-12

**Authors:** Eli Raneses, Colin F. Nolan, Rudy F. Schmiedecke, W. Hugh Lyford

**Affiliations:** a Department of Dermatology, Naval Medical Center San Diego, San Diego, California; b Department of Dermatology, Naval Hospital Camp Lejune, North Carolina

**Keywords:** autoimmune blistering disease, biologics, biologic treatment, dupilumab, epidermolysis bullosa acquisita

## Introduction

Epidermolysis bullosa acquisita (EBA) is an autoimmune blistering disease driven by antibodies to collagen VII. EBA is rare, with an estimated incidence of <1 per 1 million persons.^1^^,^^2^ Symptomatically, these patients present with tense blisters and bullae favoring sites of friction, as well as pruritus, which is often more generalized. Given its rarity, treatment recommendations are primarily based upon small cohort studies, case reports, and consensus statements.^2^^,^^3^ First-line treatment is usually topical or systemic corticosteroids, used alone or in conjunction with other immunosuppressive medications. EBA can be resistant to treatment, and some studies report remission rates around 45% over 6 years.^2^^,^^4^ Often considered in the differential diagnosis, bullous pemphigoid (BP) is another autoimmune blistering disease with autoantibodies to components of the basement membrane as well. Pruritus is an expected but not necessarily reported symptom in BP as well. Given similarities between EBA (especially the inflammatory type) and BP, we treated a patient with recalcitrant EBA with dupilumab, resulting in significant symptomatic improvement.

## Case report

**Fig 1:**
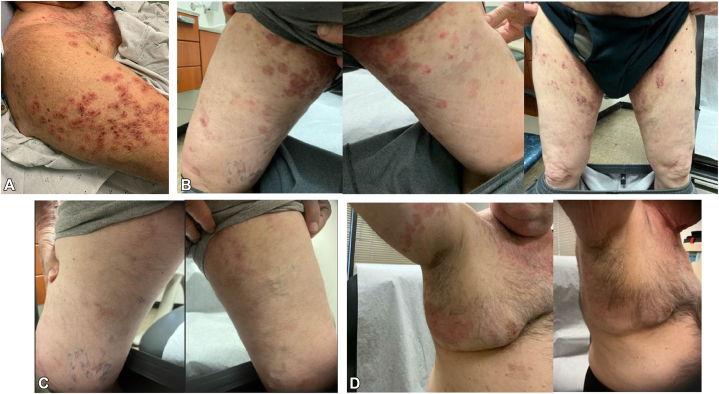
Patient photographs demonstrating. **A,** Initial epidermolysis bullosa acquisita presentation. **B,** EBA flare. **C,** Disease control with 12 months of dupilumab treatment. **D,** Patient with EBA flare of the lateral torso and disease control of the same area with dupilumab treatment.

A 65-year-old man with a 2-year history of EBA with recurrent tense bullae primarily of the medial arms, lateral torso, medial upper legs, and buttocks presented for management. He had no other history of skin disease prior to presenting with EBA, including no history of atopy, eczema, or prurigo. On initial presentation, he had had diffuse body involvement (Fig 1, *A*), with a skin biopsy demonstrating a subepidermal vesicle with neutrophils and eosinophils and direct immunofluorescence demonstrating IgG and C3 antibody deposition along the basement membrane. Diagnosis of EBA was confirmed with serology, which showed elevated autoantibodies to collagen VII, indirect immunofluorescence (IIF) consistent with EBA, and negative for antinuclear antibody, anti-BPAG1, and anti-BPAG2.

Despite continued therapy with prednisone, mycophenolate mofetil, colchicine, and topical corticosteroids, he continued to have new blister formation weekly with intense pruritus (Fig 1, *B* and *D*). As the patient’s symptoms were recalcitrant to other treatments, we discussed the risks, benefits, and alternatives to starting biologic therapy. After discussion, the patient wished to initiate off-label treatment with dupilumab. He was tapered off prednisone and mycophenolate mofetil and cross-titrated onto dupilumab. He continued dupilumab, colchicine, and topical corticosteroids for 12 months, without any blister formation for 9 months (Fig 1, *C* and *D*). He additionally reported significant improvement in pruritus. He denied any significant adverse side effects from dupilumab use. Of note, although the patient symptomatically improved, antibody titers to collagen VII have remained elevated, although his IIF has become negative (Table I). As of the patient’s most recent follow-up he has had sustained disease control on dupilumab; colchicine has been discontinued for 2 months, without new blister formation for 12 months.

**Table I:** Patient laboratory values and associated symptoms over time

Patient labs and symptoms	2023 (Presentation and flare)	2024 (Prior to dupilumab initiation)∗	2025 (2 mo after dupilumab start)†	2025 (9 mo after dupilumab start)
Collagen VII titers (U/mL)	34	40	42	44
IgG4 IIF-salt split skin	1:20	1:10	1:10	0
Symptoms	Extensive blisters	Weekly blisters, pruritus	Reduction of blisters, improved pruritus	No blisters, further improved pruritus

IIF, Indirect immunofluorescence.

∗ Patient taking high dose systemic corticosteroids, mycophenolate mofetil, and colchicine at time of laboratory draw.

† Patient on dupilumab and colchicine treatment only.

## Discussion

This case demonstrates significant symptomatic improvement in a patient with EBA treated with dupilumab associated with a conversion of the patient’s IIF positivity but without a decrease in EBA-associated antibody titers.

Dupilumab is now approved for the treatment of BP.^5^ Though EBA and BP are different diseases, they do share some similar characteristics. According to the reported results of the pivotal trials for dupilumab for BP, patients treated with dupilumab had significantly reduced itch, as well as a sustained statistically significant reduction in blisters. We performed a narrative literature review and found 2 other indexed published case reports of dupilumab being used to treat EBA with success.^6^^,^^7^ The first case presents a 51-year-old woman with recalcitrant EBA, which did not fully respond to prednisolone, azathioprine, and 2 cycles of rituximab, who successfully responded to dupilumab with no recurrence of symptoms for 12 months.^6^ The second case is of a 37-year-old woman with EBA treated with prednisolone and intravenous immunoglobulin who was able to obtain disease control with dupilumab and prednisolone but did have a recurrence in the setting of discontinuation of dupilumab, requiring continued systemic steroid and dapsone use.^7^ This case report adds to the literature another successful use of dupilumab to manage EBA, with a reduction in blister formation as well as significant improvement in pruritus. This case is uncommon because the patient did not receive infusion treatments, and we have been able to trend the patient’s IIF and autoantibody titers over time. Interestingly, our patient experienced significant symptomatic improvement in concert with a conversion from positive to negative IIF. That this patient’s symptomatic improvement and IIF conversion were not accompanied by a reduction of serum antibody titers suggests that dupilumab may impact disease course through a mechanism that does not result from a decrease in pathogenic antibody production. Larger studies should be conducted to further demonstrate the benefits and limits of dupilumab as a treatment of EBA, particularly dosing and treatment length. Further studies may be able to further elucidate the underlying mechanism for dupilumab’s apparent ability to not only improve symptoms but possibly change the disease course.

## Conflicts of interest

I (ER, CN, RS, WHL) am a military service member or federal/contracted employee of the US government. This work was prepared as part of my official duties. Title 17 USC 105 provides that copyright protection under this title is not available for any work of the United States Government. Title 17 USC 101 defines a US Government work as work prepared by a military service member or employee of the US Government as part of that person’s official duties. The remaining authors have no financial interests or conflicts of interest to disclose.
